# Bifurcation and Frequency Properties of S‑Type Neuronic Oscillators

**DOI:** 10.1021/acs.jpclett.5c00288

**Published:** 2025-04-03

**Authors:** Juan Bisquert, Roberto Fenollosa, Alicia Cordero, Juan R. Torregrosa

**Affiliations:** † Instituto de Tecnología Química (Universitat Politècnica de València-Consejo Superior de Investigaciones Científicas), Camino de Vera s/n, 46022 València, Spain; ‡ Instituto de Matemática Multidisciplinar, 16774 Universitat Politècnica de València, Camino de Vera s/n, 46022 València, Spain

## Abstract

Oscillators are nonlinear elements or systems that generate periodic signals in an electronic circuit. In neuromorphic circuits, oscillators are used to replicate essential neural processes, such as synchronization, spiking, and rhythmic activity. To obtain these functions, a broad range of systems is investigated for artificial neurons, such as electrochemical autocatalytic systems, organic electrochemical transistors, and binary oxides memristors with an insulator–metal transition. The general features of oscillators controlled by a single internal physical variable, which produces an S-type current–voltage curve with a negative differential resistance, with matched external *R* and *C* elements, are discussed. The paper provides a classification of dynamical behaviors that will be found in the practical investigation and applications. A Hopf bifurcation ensures the existence of a limit cycle where the oscillations have small amplitude and nearly sinusoidal form. Slower relaxation oscillations occur at large values of the capacitor time constant since the internal variable becomes very fast in comparison to the variation of voltage. The values of the oscillation frequencies across the whole variation of bifurcation parameters are described, which facilitates the application of the oscillators in coupled configurations such as oscillating neural networks.

## Graphical abstract

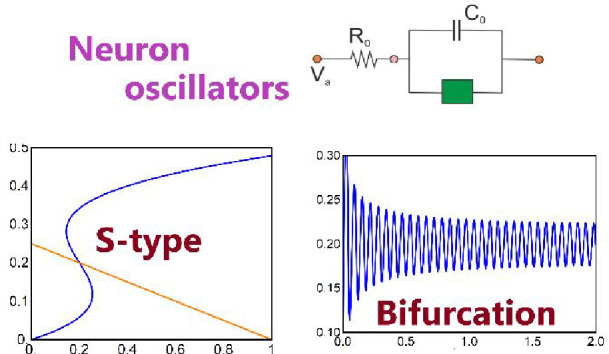

## 1 Introduction

Neuromorphic circuits are inspired by the structure and functionality of biological neural systems, aiming to mimic the efficiency and adaptability of the brain in computing tasks such as pattern recognition, sensory processing, and decision-making.^1–4^ A critical component of neuromorphic systems is the oscillator, which plays a central role in emulating the rhythmic and temporal dynamics of biological neural networks. Oscillators are nonlinear elements or systems that generate periodic signals in an electronic circuit. In neuromorphic circuits, oscillators are used to replicate essential neural processes, such as synchronization, spiking, and rhythmic activity. These properties are key to tasks like temporal encoding, coordination of neural populations, and energy-efficient signal processing.

Oscillatory devices have recently garnered considerable attention as essential elements in computing systems inspired by biomimetic neuronal spiking. The use of oscillators in neuromorphic circuits offers several advantages. They can facilitate communication between different parts of the circuit, enhance the robustness of neural representations, and enable low-power computing. Recurrent artificial neural networks are an important computational paradigm capable of solving several optimization problems.^5^ There is extensive research of neural network computational applications using these types of oscillators.^1,6–15^ Understanding the time scales that govern this spiking is crucial for designing fast, controllable, and energy-efficient devices. A suitable formulation of this computing paradigm is an oscillating neural network (ONN), in which information is encoded in the phase relationships between coupled oscillators.^16,17^ For computing with coupled oscillators,^16,18,19^ the frequency and phase of the oscillators become a topic of great interest.^8,20^ In this paper the oscillation frequency and the bifurcation characteristics of nonlinear neuromorphic oscillators are described.

We focus our attention on analog oscillators based on a physical system that contains a multivalued current–voltage curve. In essence, the physical model of a neuron-like oscillator is based on a conduction current that depends at least on two variables: the voltage and an internal state variable *x*. The latter causes resistive switching with a negative differential resistance (NDR) sector. In general the oscillators can be classified as N-shape or S-shape, depending on the type of negative resistance feature.^21–24^ Compact oscillators are formed by connecting a two-terminal nonlinear device with an electrical resistor and a capacitor.^23^ The system then has a dynamical slow-fast combination that enables oscillations. The periodic spiking often occurs suddenly under a variation of parameters such as the external capacitance or the series resistance, in the process of a Hopf bifurcation.^25,26^

Emerging technologies, such as threshold switching memristors^27–30^ and organic transistors,^31–36^ have expanded the possibilities for designing tunable, and energy-efficient oscillators tailored to neuromorphic applications. Binary oxides memristors as VO_2_ have been extensively researched.^7,9^ In NDR electrochemical systems, the interplay between charge transfer and surface species leads to oscillatory behavior. Electrochemical oscillators have been studied for many decades.^21,37–42^ These types of devices are excellent candidates for neurons in neuromorphic circuits, and their coupling properties present promising properties for the construction of ONN.^7,43–46^

In the context of these applications, here we show an analysis of properties of bifurcation and frequency of oscillations of a single S-type oscillator, complementing previous description of the N-type systems.^26,47,48^ Here, specific physical mechanisms, which have been widely reviewed,^49–51^ are not addressed, although some specifical systems are described summarily in Section 5. We present a general model of an oscillator containing a single degree of freedom in the resistive switching, and obtain the properties of the nature of oscillations, from harmonic variation close to the Hopf bifurcation point, to the slower relaxation oscillations. We show explicitly the variation of frequency and period in terms of the parameters of the circuit. The classification of behaviors that occur for any type of single-variable S-type oscillators will facilitate the practical investigation and applications of these systems.

## 2 Introduction to Oscillating Systems

As an introductory example we show the FitzHugh-Nagumo (FHN) neuron model.^52,53^ It is one of the simplest models with nontrivial behavior that enables a phase plane analysis^54–57^ and it is used in a multitude of neural oscillators.^58–60^ This model is representative of a wider class of N-type oscillators.^26,47,48,61^ The S-type oscillator, that is the topic of this work, will be discussed in the remaining sections of the paper. Based on FHN model, we introduce the methods and properties of dynamical oscillatory systems that are common to the different types of physical oscillators.^62–65^

### 2.1 The FitzHugh-Nagumo Model

As pointed out by Nagumo et al.,^53^ the FHN model can be represented by the electrical model shown in Figure 1. In standard analysis of a single neuron we write *I*_*c*_ = 0, as there is no external coupling. We assume that the system is current-driven, *E*_0_ = 0, hence the variables are the voltage *u* and the inductor current *i*_*L*_, and the source current is a parameter. Hereafter we denote the internal variable *x* = *i*_*L*_. The circuit is shown in Figure 1b and it is governed by the equations^60,66^

1 $$C_{0}\frac{du}{dt}=I_{0}-I_{G}\left(u\right)-x$$

2 $$L_{a}\frac{dx}{dt}=u-R_{a}x$$

The FHN model is defined by the variable resistor of the form

3 $$I_{G}\left(u\right)=\frac{1}{R_{0}}\left(\frac{u^{3}}{3}-u\right)$$

where *R*_0_ is a constant resistor parameter.

**Figure 1:**
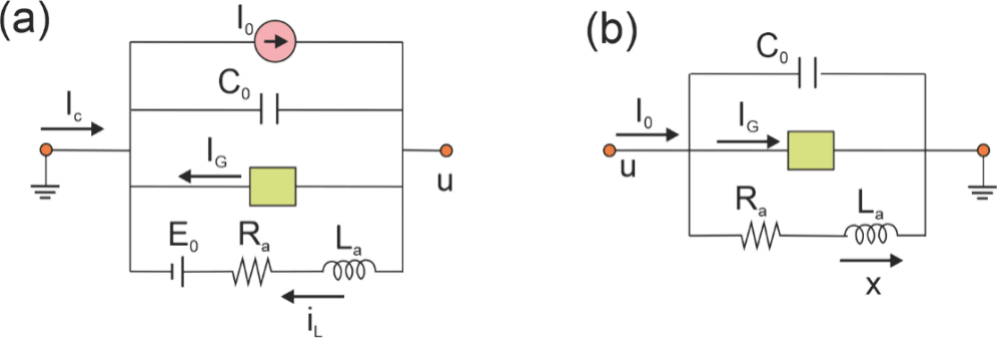
(a) Electrical representation of the FHN model. *u* voltage; *I*_*c*_ external coupling current; *I*_0_ internal source current; *i*_*L*_ current in the inductor; *I*_*G*_(*u*) variable resistor; *C*_0_ capacitance; *E*_0_ voltage source; *R*_*a*_ resistor; *L*_*a*_ inductor. (b) Simplified circuit.

The system is characterized by the time constants for the capacitive *R*_0_ *C*_0_ and inductive *R*_*a*_ *L*_*a*_ subcircuits

4 $$\tau_{0}=R_{0}C_{0}$$

5 $$\tau_{L}=\frac{L_{a}}{R_{a}}$$

In applications of neuron dynamics,^67^ the interpretation of the variables is the membrane voltage *u*, the transmembrane current *I*_0_ and an internal recovery current *x*, that represents the changes in ion-channel conductance as a function of the voltage. In addition, the voltage response time is τ_0_ and the recovery current response time is τ_*L*_. One can rewrite the above eqs 1 and 2 in the convenient form^55,58^

6 $$\tau_{0}\frac{du}{dt}=u-\frac{u^{3}}{3}+R_{0}\left(I_{0}-x\right)$$

7 $$\tau_{L}\frac{dx}{dt}=\frac{u}{R_{a}}-x$$

Sometimes a different parametrization uses the form *bx* in eq 7, with *b* > 0.^47^ The stationary solution of eqs 6, 7 is

8 $$I_{0}=\frac{1}{R_{0}}\left(\frac{u^{3}}{3}-u\right)+\frac{u}{R_{a}}$$

This is the blue line in Figure 2a, which includes, in addition, the plot of the two main components (green and pink curves) contributing to *I*_0_. For a specific applied current, eq 8 gives a steady-state point (*u̅*,*x̅*) along the blue line, which can be stable or unstable, as discussed later. The blue dot shown in Figure 2a, b and c is an example for *I*_0_ = 0.507 A (horizontal dashed orange line), (*u*_*app*_ = 0.7 *V*). We observe in Figure 2c, d that in this case the system leads to oscillatory behavior as explained below.

**Figure 2:**
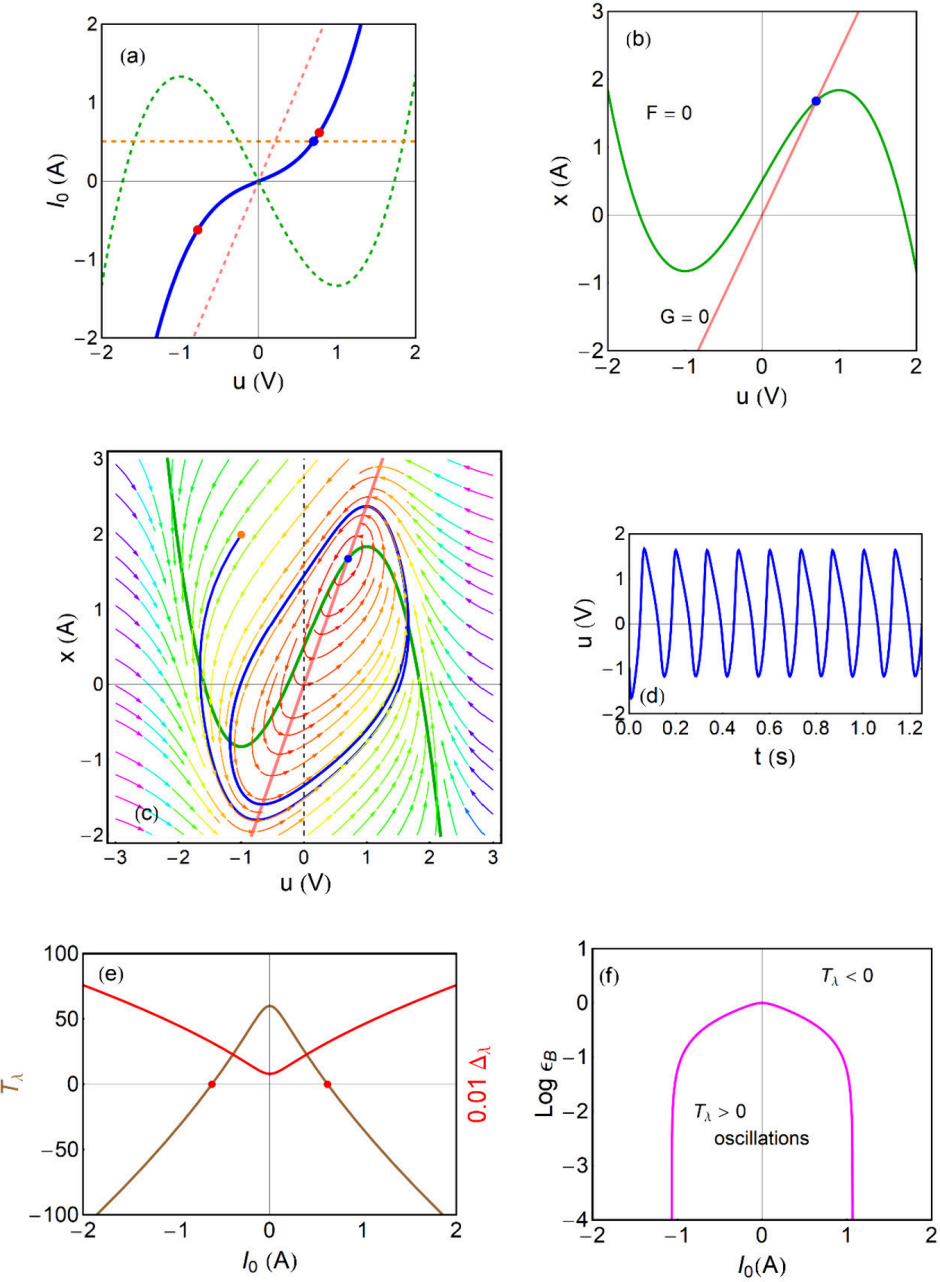
FitzHugh-Nagumo model. (a) *u*–*I*_0_ plane. The green line is the fast component of the current, and the pink line is the slow one. When added, they give the blue line that is the stationary in the *I*_0_–*u* characteristic. The blue point is the steady-state point at the nominal potential (*u*_*app*_ = 0.7), and the orange line is the corresponding constant current *I*_0_ = 0.507 *A*. The red points are the Hopf bifurcations. (b) Nullclines in *u*–*x* plane. (c) Nullclines, trajectories and vector velocities at constant current *I*_0_ = 0.507 *A*. The orange point is the starting condition. Color streamlines indicate the norm of the vector field. (d) Voltage evolution with time. (e) Trace and determinant. (f) Bifurcation diagram. Parameters: *R*_0_ = 0.5 Ω; *R*_*a*_ = 0.417 Ω; τ_0_ = 0.01 *s*; ϵ = 0.4. *u* in V, *I*_0_, *x* in A.

### 2.2 Stable Oscillations

Let us consider a system of coupled differential nonlinear equations:

9 $$\frac{du}{dt}=F\left(u,x,I_{0}\right)$$

10 $$\frac{dx}{dt}=G\left(u,x\right)$$

Here *u* is the voltage in the device and *x* is an internal state variable, as in FHN model and in memristor models.^68–70^ *F* and *G* are multivalued nonlinear functions, and the current *I*_0_ is a parameter. In the case of the FHN model we have

11 $$F=\frac{1}{\tau_{0}}\left(u-\frac{u^{3}}{3}\right)+\frac{1}{C_{0}}\left(I_{0}-x\right)$$

12 $$G=\frac{1}{\tau_{L}R_{a}}u-\frac{x}{\tau_{L}}$$

Instead of calculating the exact solutions of the system eqs 9, 10, dynamical methods provide their asymptotic behavior by analyzing the steady state of the problem and its long-term performance.^62,64^ By solving the algebraic system

13 $$F\left(u,x,I_{0}\right)=0$$

14 G(u,x)=0

the steady-state (*u̅,x̅*) of eqs 9, 10 is found, as in eq 8. Both curves describing eqs 13, 14 are called nullclines. The nullclines give a relationship between the variables of the system where there is no temporal change of a specific variable, hence they are the points of zero velocity.^71^ Nullclines of FHN model are shown in Figure 2b, and Figure 2c shows that the flow vector in the phase portrait *u*–*x* is either vertical or horizontal at the nullclines. Note that the nullclines eqs 13, 14 do not depend on values of the capacitor *C*_0_ or inductor *L*_*a*_.

To classify the stability of a stationary point (*u̅,x̅*), the system eqs 9, 10 must be linearized for the small displacement variables X = [*u̅,x̅*]^*T*^, which gives the differential equation

15 $$\frac{dX}{dt}=JX$$

in terms of the Jacobian matrix evaluated at (*u̅,x̅*),

16 $$J=\left[\begin{array}{ll} F_{u}\left(\mathrm{u̅},\mathrm{x̅},{\mathrm{I̅}}_{0}\right) & F_{x}\left(\mathrm{u̅},\mathrm{x̅},{\mathrm{I̅}}_{0}\right)\\ G_{u}\left(\mathrm{u̅},\mathrm{x̅},{\mathrm{I̅}}_{0}\right) & G_{x}\left(\mathrm{u̅},\mathrm{x̅},{\mathrm{I̅}}_{0}\right) \end{array}\right]$$

defined by the partial derivatives *F*_*u*_ = *∂F/∂u*,...

The stability of the steady-state point depends on the eigenvalues λ of the matrix *J*, obtained by solving the quadratic equation

17 $$\lambda^{2}-T_{\lambda }\lambda +\Delta_{\lambda }=0$$

where *T*_λ_ is the trace of *J*

18 $$T_{\lambda }=F_{u}+G_{x}$$

and Δ_λ_ is its determinant.

19 $$\Delta_{\lambda }=F_{u}G_{x}-F_{x}G_{u}$$

The solution of eq 17 is

20 $$\lambda_{\pm }=\frac{1}{2}\left[T_{\lambda }\pm {\left(T_{\lambda }^{2}-4\Delta_{\lambda }\right)}^{1/2}\right]$$

As it appears in Figure 3, a stationary point satisfying Δ_λ_ > 0 is called (asymptotically) stable if *T*_λ_ < 0. Then all the close solutions are attracted by this point. It is unstable when *T*_λ_ > 0, in this case all the near solutions are repelled by it. In both cases, the steady-state point is called hyperbolic.

**Figure 3:**
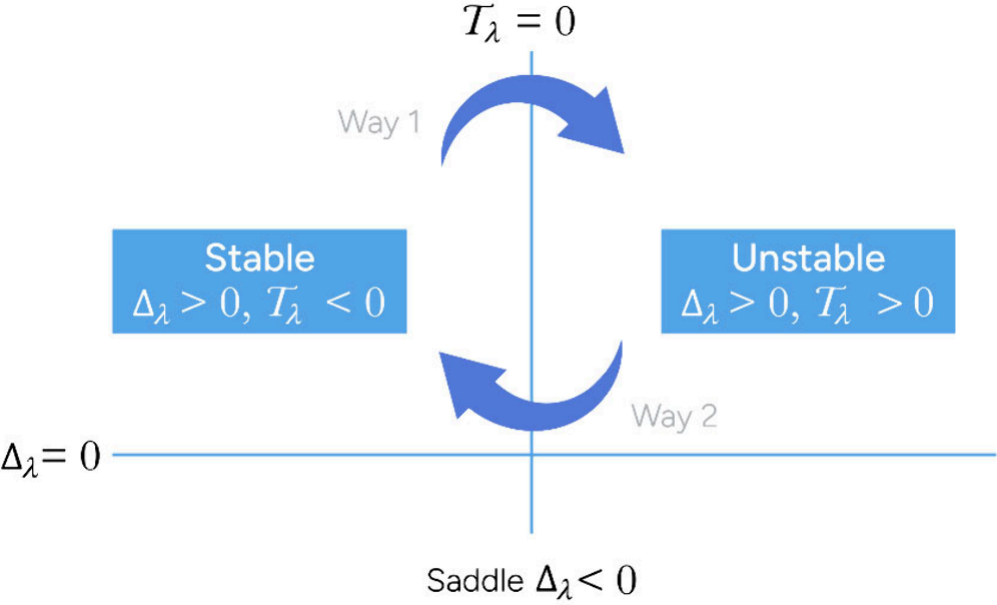
Trace-determinant characterization of steady-state points. Way 1 implies the appearance of a stable limit-cycle; in Way 2, the limit-cycle is unstable.

In some cases, the value of the parameters involved in the system determine qualitative changes in the stability: these are called bifurcation values, and the parameter is named as bifurcation parameter. A relevant bifurcation in chemistry and physics is Hopf bifurcation; in it, the stability of the stationary state changes and a periodic orbit or limit cycle is born, that is a closed and isolated trajectory in the phase portrait, leading the system to oscillation,^72,73^ see Figure 4 and Figure 2c, d.

**Figure 4:**
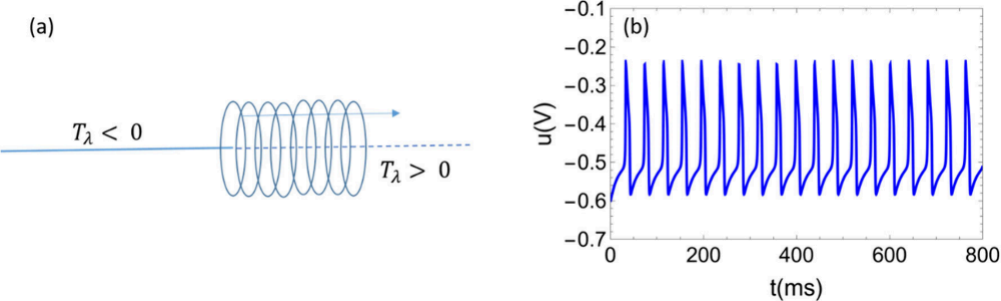
(a) Hopf bifurcation along the sign of the trace in the linearized system. (b) Spiking neuron in a Hopf bifurcation.

Hopf bifurcation appears when the real part of the eigenvalues of the Jacobian matrix *J* are null, that is, when *T*_λ_ = 0 (that is, the steady-state point becomes nonhyperbolic), while the determinant is positive. At the Hopf bifurcation the real part of the eigenvalue changes sign from negative to positive by the variation of a parameter, Figure 2e.^74,75^

The periodic orbit emerging from a Hopf bifurcation is a closed loop in the state space *u*–*x* and can be stable or unstable. In the first case (Way 1 in Figure 3), the stationary point is stable (when *T*_λ_ < 0) and, for a specific value of the bifurcation parameter, the trace becomes null *T*_λ_ = 0, and an attracting limit-cycle appears; simultaneously, the steady-state point becomes unstable (see Figure 4a). This is the desirable performance for obtaining an oscillating solution, Figure 4b. The uniqueness theorem says that solutions of eqs 9, 10 in phase space never intersect and Poincaré-Bendixon Theorem^64,76^ establishes that a solution is bounded by a closed region that does not hold any stationary point. Then the solution must approach a closed orbit, independently of the initial conditions.

If the stability of the stationary point passes from unstable (when *T*_λ_ > 0) to stable (*T*_λ_ < 0) through bifurcation (Way 2 in Figure 3), the limit-cycle that appears is unstable and the close solutions will not approach to it.

A Hopf bifurcation requires at least two variables, as in eqs 9, 10, a fast destabilizing variable and a slow stabilizing variable,^25^ usually called a slow-fast dynamical system.^77–79^ It also requires a negative differential resistance behavior in the destabilizing variable. Models formed with the two essential oscillating variables find a wide range of applications.^54,80,81^ For example, electrochemical oscillations require a fast positive feedback mechanism and a slow inhibitor one.^21,37,38,42^ The positive feedback variable is typically either the electrode potential (*E*) or the surface coverage of adsorbed species (θ), as further explained in Section 5. Many VO_2_ oscillator devices can usually be described by only two differential equations.^82,83^ However, other systems require a larger number of memory variables. Electrochemical oscillators often require three memory variables giving four differential equations,^37,84^ the same number as the Hodgkin-Huxley (HH) model, that serves as the primary mathematical framework for describing the operation of natural neurons.^72,85,86^ For simplicity, we restrict the discussion to dynamical systems with one internal state variable, *x*, which combined with the voltage *u* gives two differential equations.

The presence of the bifurcation and the negative resistance can be observed by impedance spectroscopy measurements in the frequency domain.^24,26,37,47,48,87,88^ The impedance method enables a study of the stability according to the sign of resistors and inductors associated with the fast and slow variables.^47^

For the FHN model in Figure 2, the stationary current is composed of two terms. The green component in Figure 2a contains a negative resistance, corresponding to the fast variable *u*. But the stabilizing slow *x* variable, with resistance *R*_*a*_, makes the total resistance of the blue line positive, hence the negative resistance is not visible in the measured current–voltage curve (it is “hidden”^89^). The bifurcation parameters are the ratios

21 $$\epsilon =\frac{\tau_{0}}{\tau_{L}}=\frac{R_{0}R_{a}C_{0}}{L_{a}}$$

22 $$r_{0a}=\frac{R_{0}}{R_{a}}$$

The trace and determinant are

23 $$T_{\lambda }=\frac{1}{\tau_{0}}\left(1-u^{2}-\epsilon \right)$$

24 $$\Delta_{\lambda }=\frac{1}{\tau_{0}\tau_{L}}\left(-1+u^{2}+r_{0a}\right)$$

These functions are shown in Figure 2e. The Hopf bifurcation points are the currents *I*_*Hopf*_ corresponding to

25 $$u_{Hopf}=\pm {\left(1-\epsilon \right)}^{1/2}$$

For values |*I*_0_| ≤ |*I*_*Hopf*_| it is *T*_λ_ ≥ 0 and the system shows limit cycle oscillations as presented in Figure 2b, c. The ϵ determines the range of voltages where oscillation occurs. The Hopf bifurcation occurs at

26 $$\epsilon_{B}\left(I_{0}\right)=1-{\mathrm{u̅}\left(I_{0}\right)}^{2}$$

If the capacitor *C*_0_ is too large (or the inductor too small), oscillations are not permitted. This property is observed in the bifurcation diagram of Figure 2f.

Another important class of bifurcation indicated in Figure 3 is the saddle-node bifurcation, in which the determinant becomes negative. In the Hopf bifurcation the oscillations begin with a finite frequency and the system is called a Type-II neuron in neuroscience, in contrast with the Type I oscillators of a saddle-node bifurcation, that begin at zero frequency.^54,90,91^

### 2.3 Relaxation Oscillations

In relaxation oscillations^92^ the system leaves and returns to a slowly varying steady state equilibrium current–voltage line by fast, sudden transitions.^79,93^ In general relaxation oscillations are limit cycles of a singularly perturbed dynamical system, and may appear in a fast-slow system of coupled differential nonlinear equations including a small parameter ϵ,

27 $$\epsilon \frac{du}{dt}=B\left(u,x,\epsilon \right)$$

28 $$\frac{dx}{dt}=G\left(u,x,\epsilon \right)$$

where *B* and *G* are multivalued nonlinear functions (*F* = *B/ϵ*), *u* is the fast variable and *x* is the slow one. The ϵ parameter usually corresponds to a ratio of intrinsic characteristic times of the two variables, eq 21. The smallness of ϵ ≪ 1 is related to the fast transitions of the oscillations.

Considering the FHN model, in Figure 5a, b we show relaxation oscillations that are obtained for ϵ = 0.01. The evolution in Figure 5a tracks the green *u̇* = 0 nullcline until a fold point is reached, and then a sudden jump occurs to the other stable branch. Since d*x*/d*t* ≪ d*u*/d*t* the jump consumes a negligible time, and the voltage cycles in Figure 5b have a sectors of constant velocity separated by vertical transitions.

**Figure 5:**
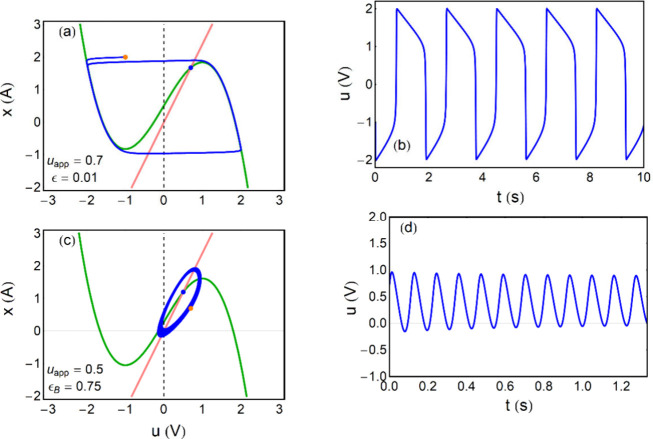
Oscillations in FitzHugh-Nagumo model. (a), (c) Evolution and nullclines in *u*–*x* plane. The blue point is the fixed point at the nominal potential. The orange point is the initial condition. (b), (d) Voltage evolution with time. Same parameters as Figure 2 with ϵ and *u*_*app*_ as indicated.

For the case of FHN in eqs 11, 12, the form eqs 27, 28 is obtained introducing a dimensionless time, *t* = τ_*L*_ *t*′:

29 $$\epsilon \frac{du}{dt^{\prime }}=u-\frac{u^{3}}{3}+R_{0}\left(I_{0}-x\right)$$

30 $$\frac{dx}{{dt}^{\prime }}=\frac{u}{R_{a}}-x$$

The F-nullcline corresponds to the curve *x* = (*R*_0_ *I*_0_ + *u* – *u* ^3^/3)/*R*_0_, obtained also for ϵ = 0 (known as singular limit), being the G-nullcline *u/R*_*a*_ – *x* = 0. In this case, the (unstable) stationary point (as intersection of both curves) is the blue point in Figure 5(a). The folding points of null rate d*x*/d*u* = 0, are respectively *p*_1_ = (−1, *I*_0_ – 2/(3*R*_0_)) and *p*_2_ = (1, *I*_0_ + 2/(3*R*_0_)). These are key values to calculate the period and frequency of the oscillations.

### 2.4 Frequency of the Oscillations

Let us define the angular frequency^20^

31 $$\omega_{0}=\Delta_{\lambda }^{1/2}$$

At the Hopf bifurcation, *T*_λ_ = 0. By eq 20 the eigenvalues take the form

32 $$\lambda_{+}=i\omega_{0},\lambda_{-}=-i\omega_{0}$$

The diagonal matrix *D* = *P* ^–1^ *J P* is

33 $$D=\left[\begin{array}{ll} \lambda_{+} & 0\\ 0 & \lambda_{-} \end{array}\right]$$

where the matrix *P* is obtained from the eigenvectors. Let *Y* = [*v, y*]^*T*^ be the coordinates defined by *Y* = *P* ^–1^ *X*. Equation 15 becomes

34 $$\frac{dY}{dt}=DY$$

and the solution of the system is

35 $$v=v_{0}e^{i\omega_{0}t}$$

36 $$y=y_{0}e^{-i\omega_{0}t}$$

The harmonic oscillation around the stationary point in phase space *u*–*x* is given by X = *PY*. The period of the oscillations, close to the Hopf bifurcation, is

37 $$T_{H}=\frac{2\pi }{\omega_{0}}$$

These oscillations are shown in Figure 5c, d.

If we move away from the bifurcation, using a smaller capacitor (downward in Figure 2f), then eqs 11, 12 produce relaxation oscillations, as already discussed. Since the time of the jump can be neglected, the method to calculate the frequency of the relaxation oscillations consists of the integration over the segments of the steady state curve on the slow variable^79,92,93^

38 $$T_{R}=\oint \frac{dt}{dx}\;dx=\oint \frac{dx}{G}$$

In order to calculate the period of the oscillation along the trajectory of the orbit (considered its fast part as instantaneous), we take into account the slow variable such that *ẋ* = *G*(*u*,*x*,*ϵ*) and then

39 $$T_{R}=\left|\oint \frac{1}{G\left(u,x,\epsilon \right)}\;dx\right|=\left|\oint \frac{1}{\frac{u}{{\tau_{L}R}_{a}}-\frac{x}{\tau_{L}}}\;dx\right|=-2\int_{1}^{2}\frac{1-u^{2}}{\frac{1}{\tau_{L}}\left[\left(\frac{R_{0}}{R_{a}}-1\right)u+\frac{u^{3}}{3}-R_{0}I_{0}\right]}\;du\approx 1.856\;s$$

being *R*_0_ = 0.5 Ω, *R*_*a*_ = 0.417 Ω and *I*_0_ = 0.507 *A*. Therefore, the frequency of the oscillations is

40 $$\omega_{R}=\frac{2\pi }{T_{R}}\approx 3.38526\;rad/s$$

## 3 S-Type Based Oscillators

Having reviewed the general features of N-type oscillators, now we turn our attention to the goal of this paper, that is oscillators that are built based on a physical device that displays a S-type current–voltage curve, which is used in effective neuron devices (see Section 5). The standard structure of such an oscillator is shown in Figure 6a.^21–23^ It consists of the nonlinear element (green), and external elements resistor *R*_0_ and capacitor *C*_0_, that enable an oscillatory voltage *u*. The *I*_0_ is the external current and *V*_*a*_ the external voltage. We always have

41 $$I_{0}=\frac{V_{a}-u}{R_{0}}$$

In general, self-sustained oscillations must be obtained when the system is placed in a stationary point that is unstable, as explained before. The fast component of N-shaped oscillators is multivalued in current, Figure 2a, while S-type oscillators (Figure 6c, d) are multivalued in voltage.^21–24^ Experimental examples of S-shaped oscillators are presented in the applications described in Section 5.

**Figure 6:**
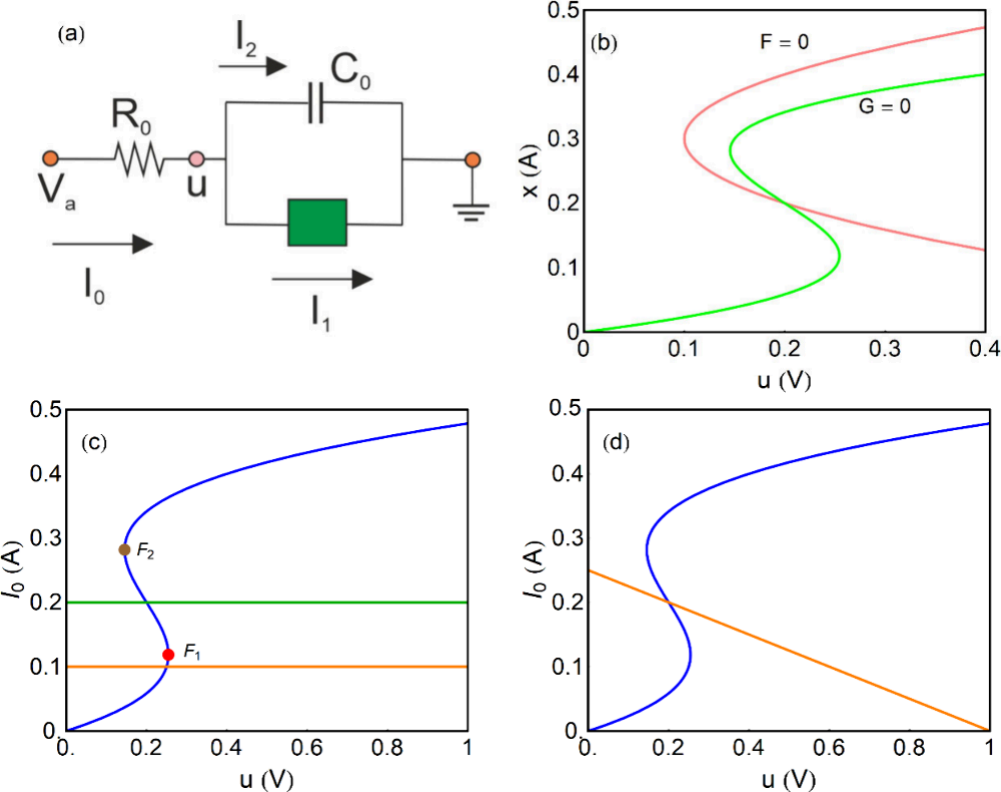
(a) Oscillatory circuit. (b) Nullclines obtained from the dynamical equations in equilibrium, *u̇* = *F* = 0 and *ẋ* = *G* = 0. (c) Stationary curve *u*(*I*_0_) (blue) and constant current lines: *I*_0_ = 0.1 *A* (orange), *I*_0_ = 0.2 (green). The folding points are indicated. (d) Stationary curve *u*(*I*_0_) (blue) and line of constant voltage *V*_*a*_ = 1 *V* (orange). Parameters *a* = 50 *V/A* ^3^, *b* = 0.2 *A*, *c* = 1 Ω, *R*_0_ = 4 Ω.

### 3.1 Current and Voltage Control Systems

It is necessary to distinguish between two modes of operation of the oscillator in Figure 6a: constant applied current *I*_0_, in Figure 6c, and constant applied voltage *V*_*a*_ in Figure 6d. Both methods have pros and cons. Voltage signals are most convenient for neuromorphic circuits, as they are widely used in traditional CMOS technology, making integration with conventional digital circuits simpler. Amplifiers and other supporting circuits are more readily available for voltage signals. Furthermore, circuits can be designed to operate at very low supply voltages, reducing overall power consumption. However, current-mode circuits can naturally emulate neural systems in biology that operate primarily with currents (e.g., ionic currents across membranes). Current-mode circuits can be highly compact because currents can be summed directly at nodes without requiring large resistors or capacitors. For biological fidelity (e.g., spiking neural networks), current-based designs might be preferable. At constant current (galvanostatic mode in electrochemistry) the external resistor in Figure 6a makes no role. Hence the fixed voltage mode (potentiostatic) contains an additional parameter, *R*_0_.

The external *R*_0_ *C*_0_ elements are essential for the oscillatory system. In electrochemistry *C*_0_ is a double-layer capacitance, and the *R*_0_ is a combination of the electrolyte resistance and an external resistance. Then a major classification of electrochemical oscillators^25^ occurs according to whether the oscillator (1) oscillates under strictly potentiostatic conditions, that is, in the absence of any ohmic potential drop across the electrolyte or across an external series resistance; (2) it oscillates only under potentiostatic conditions in the presence of an ohmic resistance (be it solution or external ohmic series resistance); and, (3) it oscillates under both potentiostatic and galvanostatic conditions.

### 3.2 Dynamical System

As mentioned before a minimal system for the occurrence of a Hopf bifurcation requires two variables, a fast-destabilizing variable and a slow stabilizing variable.^25,26^ Therefore, to obtain an oscillatory system we need at least two dynamical equations. The first one is obtained from the addition of currents in Figure 6a:

42 $$I_{0}=C_{0}\frac{du}{dt}+I_{1}$$

The second equation is due to an internal variable *x* that determines the conductance of the nonlinear element. In binary metal oxides this variable originates from an electronic order breakdown (defect alignment forming a conducting filament) or from a temperature-dependent transport effect, such as Poole–Frenkel conduction, and local Joule heating.^94–99^ To complete the model a relaxation equation for d*x*/d*t* is needed.^82^

### 3.3 An S-Type Model

Since the current is controlled by the internal variable *x*, we can assume that the conductance of the nonlinear element depends on *x* as follows

43 $$I_{1}=g\left(x\right)u$$

The *g*(*x*) is the physical function that fixes the specific model. Let us introduce the time dependence of *x* by the equation

44 $$\tau_{k}\frac{dx}{dt}=g\left(x\right)u-x$$

Here, τ_*k*_ is the relaxation time for the transition of *x* between the two conductance branches.

Equations 42 and 44 form the typical dynamical structure of a memristor.^68–70^ For producing oscillations we further need: (a) the parallel capacitor; (b) the physical memristor needs to include a negative resistance sector as shown in Figure 6c.^26^ The necessity for this feature is discussed later.

The dynamical system eqs 42, 44 has the general expression eqs 9, 10. However, note that eqs 42, 44 do not need the addition of an inductor, in contrast to the FHN model in Figure 1. This is because the relaxation eq 44 of the nonlinear conductor already has the structure of a chemical inductor.^47,100^

Equations 42 and 44 can be expressed in terms of functions *F, G* as follows:

45 $$\frac{du}{dt}=F=\frac{1}{C_{0}}\left(I_{0}-g\left(x\right)u\right)$$

46 $$\frac{dx}{dt}=G=\frac{1}{\tau_{k}}\left(g\left(x\right)u-x\right)$$

In the dynamical equations we use *I*_0_ or *V*_*a*_, applying eq 42, according to the desired operation mode.

Let us consider first the operation at constant current, eq 45. For *u̇* = *F* = 0 the nullcline is

47 $$u=\frac{I_{0}}{g\left(x\right)}$$

For *ẋ* = *G* = 0 it is

48 $$u=\frac{x}{g\left(x\right)}$$

The stationary point is at the intersection of the nullclines, at *x* = *I*_0_, and we have *I*_1_ = *I*_0_.

Let us consider the steady state characteristics at *u̇* = *F* = 0 and *ẋ* = *G* = 0. In the S-type oscillators *u*(*I*_0_) is single valued. The differential resistance is

49 $$R_{d}=\frac{du}{dx}=\frac{1-g^{\prime }\left(x\right)u}{g\left(x\right)}$$

The region of NDR is when *g*′(*x*)*u* > 1 and *g*(*x*) > 0. The condition d*u*/d*x* = 0 defines the folding points (*u*(*x*_*F*_),*x*_*F*_) of the current–voltage curve. They satisfy

50 $$g^{\prime }\left(x_{F}\right)u\left(x_{F}\right)=1$$

To illustrate a characteristic S-shape current–voltage curve a cubic function is adopted of the type^24,83^

51 $$u=a\left[{\left(I_{0}-b\right)}^{3}+b^{3}\right]-cI_{0}$$

in terms of parameters *a*, *b*, *c*. This is the blue curve in Figure 6c, that indicates steady state points when the system is measured at fixed current. The conductance function is

52 $$g\left(x\right)=\frac{1}{a\left(x^{2}-3bx+3b^{2}\right)-c}$$

The phase portrait showing the nullclines is presented in Figure 6b. Equation 47 gives the stationary current–voltage curve, *u*(*I*_0_), shown in the blue curve in Figure 6c, that matches the green nullcline in Figure 6b. The current turning (fold) points have the values

53 $$I_{F1}=b+{\left(\frac{c}{3a}\right)}^{1/2}$$

54 $$I_{F2}=b-{\left(\frac{c}{3a}\right)}^{1/2}$$

They are shown in Figure 6c. The associated voltage points are *u*_*F*1_ = *u*(*I*_*F*1_), *u*_*F*2_ = *u*(*I*_*F*2_).

We remark that eq 44 is a standard equation for a memristor, but more complex nonlinear expressions are used depending on the material framework.^101–103^ The eq 52 cubic expression provides the necessary S-shape in the simplest mathematical form.

## 4 Characteristics of Bifurcation and Oscillations

### 4.1 Bifurcation Conditions under Constant Current

To analyze the bifurcation properties that establish the range of oscillatory domains, a linear stability analysis is considered, as explained before.^62,65^ Equations 45 and 46 are expanded around a stationary point (*u̅,x̅*) at *F* = *G* = 0. From now on we write *u* = *u̅*, *x* = *x̅*. We get the Jacobian matrix

55 $$J=\left[\begin{array}{ll} -\frac{g\left(x\right)}{C_{0}} & -\frac{g^{\prime }\left(x\right)u}{C_{0}}\\ \frac{g\left(x\right)}{\tau_{k}} & \frac{1}{\tau_{k}}\left(g^{\prime }\left(x\right)u-1\right) \end{array}\right]$$

and expressions of the trace

56 $$T_{\lambda }=-\frac{g\left(x\right)}{C_{0}}+\frac{1}{\tau_{k}}\left(g^{\prime }\left(x\right)u-1\right)$$

and the determinant

57 $$\Delta_{\lambda }=\frac{g\left(x\right)}{C_{0}\tau_{k}}$$

The determinant is always positive, Δ_λ_ > 0. The bifurcation region with respect to (*I*_0_, *C*_0_) is given by *T*_λ_ = 0, and the limit cycle oscillations occur for *T*_λ_ > 0. We observe in eq 56 that the only source of positive *T*_λ_ is the term in *g*′. This is the reason why the negative resistance (*g*′(*x*)*u* > 1, *g*(*x*) > 0) is necessary. The bifurcation can happen only when the current is between the folding points. On another hand, if the capacitor *C*_0_ becomes small the first term in eq 56 prevails and the oscillations cannot happen. This result indicates a lower bound to the value of the capacitor *C*_0_ that produces oscillations, as remarked in the literature.^8,83^ The value of the capacitor at the bifurcation is

58 $$C_{0B}=\tau_{k}\frac{g\left(x\right)}{g^{\prime }\left(x\right)u-1}$$

The bifurcation diagram is shown in Figure 7a. This diagram is experimentally observed in electrochemical oscillations, as shown in Figure 7b.^84,104^ Note that at the fold points *u*_*F*1_, *u*_*F*2_ in Figure 7a the capacitance *C*_0B_ → ∞, by eq 58. Figures 7c, d show the bifurcation regions with respect to both *I*_0_, *C*_0_, and the amplitude of the oscillations.

**Figure 7:**
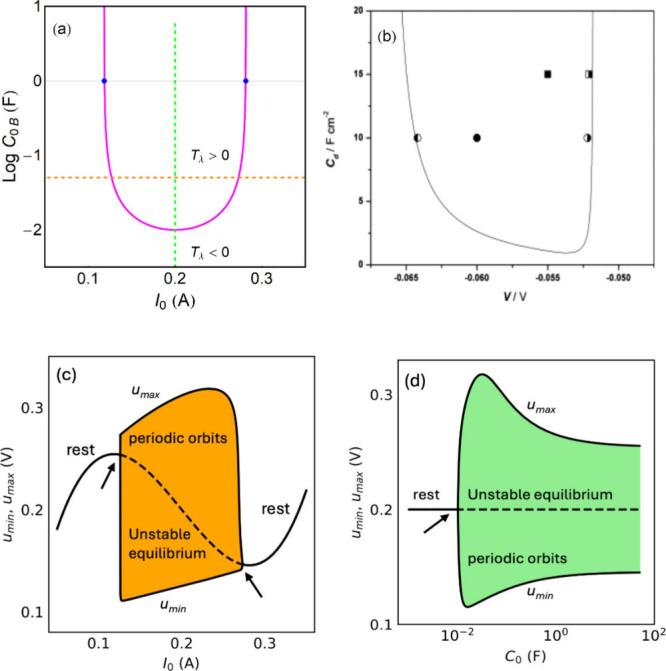
Bifurcation characteristics for constant applied current *I*_0_. (a) The pink line is the value of capacitance that gives *T*_λ_ = 0. The blue points are the folding points of the *u*(*I*_0_) curve. The vertical and horizontal dashed lines correspond to the projection lines of constant *I*_0_ (green) and constant *C*_0_ through which calculation of the maximum and minimum *u* values has been performed in (c) and (d), respectively. The arrows indicate the points at which Hopf bifurcation occurs. (b) Bifurcation diagram of the Lee–Jorné model for Zn electrodeposition. Solid curve shows the locus of Hopf bifurcation in the specific double-layer capacitance *C*_*d*_ (F cm^–2^) vs circuit potential *V* (V) parameter space. Symbols identify the special points where synchronization of two oscillators was achieved. Reprinted from ref 104 with permission from Elsevier.^104^ The parameters for the simulations are *a* = 50 *V/A* ^3^, *b* = 0.2 *A, c* = 1 Ω,τ_k_ = 0.01 *s*.

### 4.2 Oscillations at Constant Current

When the parameters *I*_0_, *C*_0_ are in the oscillation region we obtain a permanent closed trajectory in the phase portrait, independent of the starting point, as shown in Figure 8a. The oscillations in a stable limit cycle occur when the *ẋ* = 0 nullcline (pink) intersects the intermediate branch of the S-shaped line *u̇* = 0 (green) in the region where d*u*/d*x* < 0. This is shown in Figure 8a and Figure 8c. In the Figure 8e the current is outside the region between the folding points and the system simply reaches a fixed point, that is asymptotically stable.

**Figure 8:**
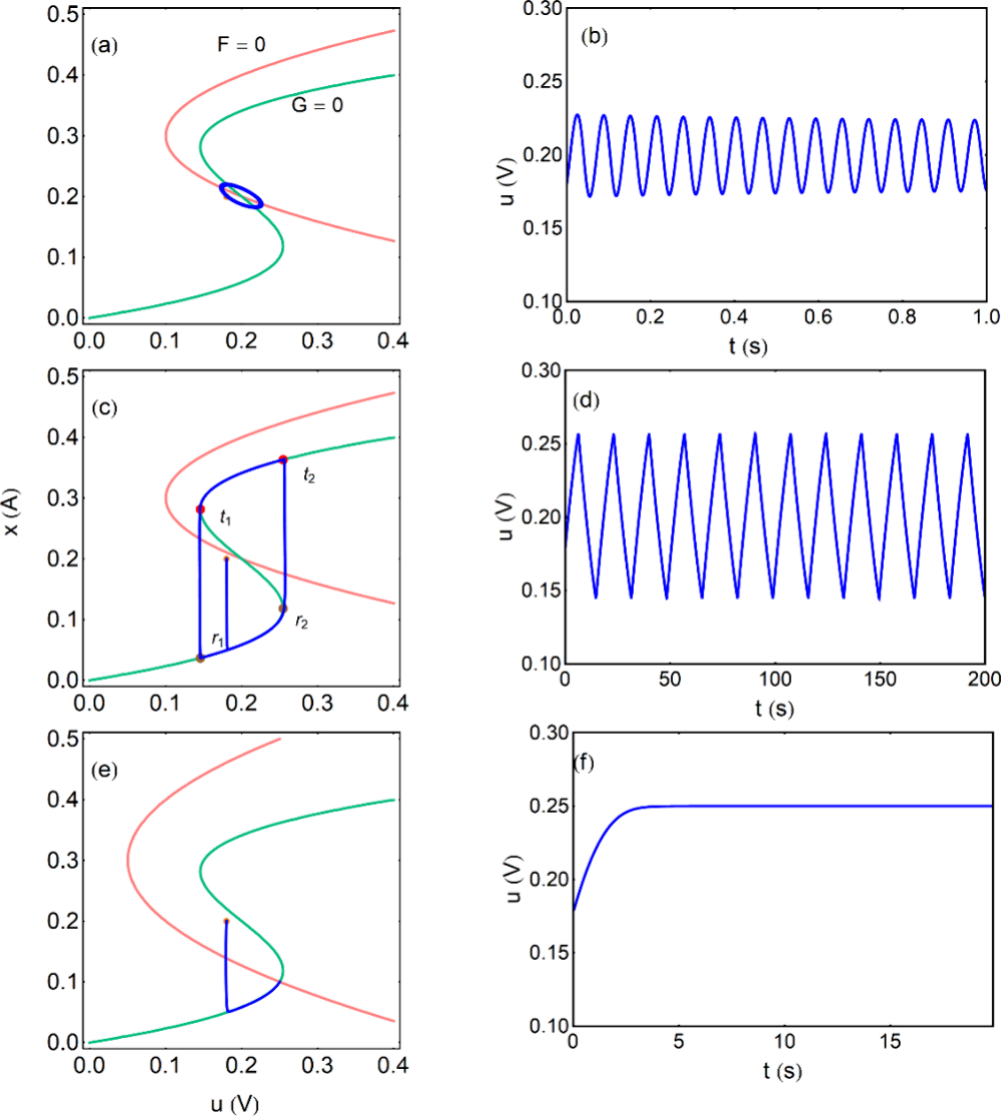
Oscillatory behavior at fixed current. Phase portrait plot, nullclines (a), (c), (e) and trajectory of *u* variable (b), (d), (f) of the dynamical evolution. The orange point is the initial condition. The points *r*_*i*_ = (*u*_*i*_ *,x*_*i*_), *t*_*i*_ = (*u*_*i*+2_,*x*_*i*+2_), *i* = 1, 2, indicate the trajectory of relaxation oscillations. Parameters *a* = 50 *V/A* ^3^, *b* = 0.2 *A*, *c* = 1 Ω, τ_k_ = 0.01 *s*, *T*_*H*_ = 2 π/Δ_λ_^1/2^. (a, b) *I*_0_ = 0.2 *A*, *C*_0_ = 0.0101 *F*, *T*_*H*_ = 0.063 *s*, (c, d) *I*_0_ = 0.2 *A*, *C*_0_ = 10 *F*, *T*_*H*_ = 2.00 *s*, (e, f) *I*_0_ = 0.1 *A*, *C*_0_ = 1 *F*. The initial conditions are *u*(*t*_0_) = 0.18 V, *x*(*t*_0_) = 0.2 A.

In Figure 8a the capacitor value is close to the Hopf bifurcation value *C*_0B_ and we find small amplitude, nearly sinusoidal oscillations, as discussed above. The frequency of oscillations is

59 $$\omega_{0}={\left(\frac{g\left(x\right)}{C_{0}\tau_{k}}\right)}^{1/2}$$

Introducing eq 58 we have the frequency at the Hopf bifurcation

60 $$\omega_{0H}=\frac{1}{\tau_{k}}{\left(g^{\prime }\left(x\right)u-1\right)}^{1/2}$$

This frequency is represented in Figure 9a. The value at *I*_0_ = 0.2 *A*, *C*_0_ = 0.01 *F*, is ω_0*H*_ = 99.9 *rad/s*, *T*_*H*_ = 0.0631 *s*, that accurately describes the sinusoidal oscillations in Figure 8b. Note that for any finite value of *C*_0_, the Hopf bifurcation frequency is finite, ω_0*H*_ > 0.

**Figure 9:**
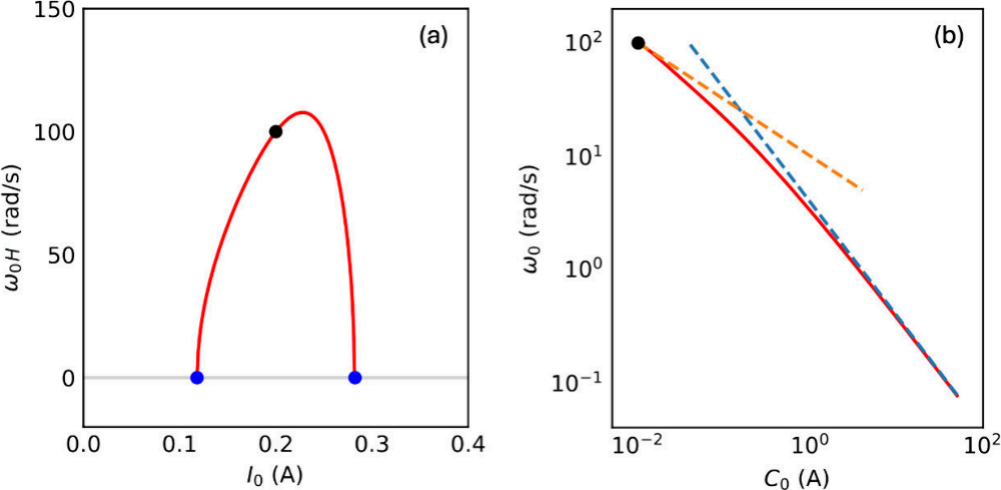
(a) Frequency of the oscillations at the Hopf bifurcation, ω_0*H*_(*C*_0*H*_,*I*_0_) with varying *I*_0_. The blue points indicate the interval limits where oscillations occur. (b) Frequency of the oscillations with varying *C*_0_ at *I*_0_ = 0.2 A. The black point in (a) and (b) corresponds to the same state at the Hopf bifurcation. The dashed orange and blue lines correspond to eqs 59 and 70, respectively. Parameters *a* = 50 *V/A* ^3^, *b* = 0.2 *A*, *c* = 1 Ω, τ_k_ = 0.01 *s*.

In Figure 9b (red line) we show the frequency of oscillations as a function of capacitance, at fixed current (i.e., following the green vertical line in Figure 7a), obtained by numerical computation. The black dot indicates the start of the oscillations. The eq 59, indicated by the orange dashed line in Figure 9b, is valid only in a small region close to the bifurcation. In this region an explicit solution to the oscillation can be obtained as follows.

Using *T*_λ_ = 0 one can write, at the bifurcation,

61 $$J=\left[\begin{array}{ll} -\frac{g\left(x\right)}{C_{0}} & -\frac{1}{C_{0}}\left(1+\frac{g\left(x\right)\tau_{k}}{C_{0}}\right)\\ \frac{g\left(x\right)}{\tau_{k}} & \frac{g\left(x\right)}{C_{0}} \end{array}\right]$$

and the *P* matrix is obtained

62 $$P=\left[\begin{array}{ll} -\frac{g\left(x\right)}{C_{0}}+i\omega_{0} & -\frac{g\left(x\right)}{C_{0}}-i\omega_{0}\\ \frac{g\left(x\right)}{\tau_{k}} & \frac{g\left(x\right)}{\tau_{k}} \end{array}\right]$$

Applying *X = PY* to eqs 35, 36 the oscillatory solution is

63 $$u=A_{0}\;\cos\left(\omega_{0}t-\phi \right)$$

64 $$x=\frac{g\left(x\right)a_{0}}{\tau_{k}}\;\cos\left(\omega_{0}t\right)$$

where *a*_0_ is a constant and the amplitude and the phase angle are

65 $$A_{0}=a_{0}{\left(\frac{{g\left(x\right)}^{2}}{C_{0}^{2}}+\frac{g\left(x\right)}{C_{0}\tau_{k}}\right)}^{1/2}$$

66 $$\phi =\mathrm{atan}\left(\frac{\omega_{0}C_{0}}{g\left(x\right)}\right)$$

These last results describe well the Figure 8a, b.

On the other hand, in Figure 8c, d it is *C*_0_ ≫ *C*_0B_ and the system produces typical relaxation oscillations of larger amplitude.^79,92,93^ The evolution tracks the green *ẋ* = 0 curve until a fold point is reached, and then a sudden jump occurs to the other stable branch. The jump consumes a negligible time, and the spikes in Figure 8d have a triangular form.

We remark that in eqs 45, 46 the *x* is the fast variable and *u* is the slow one. In the relaxation oscillations of Figure 8c, d the system moves slowly along the *G* nullcline. Thus, the role of the variables is reversed with respect to the FitzHugh-Nagumo model in eqs 11, 12.

The period *T*_*R*_ is obtained as indicated in eq 38, using eq 45,

67 $$T_{R}=\oint \frac{du}{F}=C_{0}\oint \frac{du}{\left|I_{0}-x\right|}=C_{0}\left(\int_{u_{1}}^{u_{2}}\frac{du}{\left|I_{0}-x\right|}+\int_{u_{3}}^{u_{4}}\frac{du}{\left|I_{0}-x\right|}\right)$$

along the segments *t*_1_–*t*_2_ and *r*_1_–*r*_2_ of the trajectory in the *G* = 0 curve indicated in Figure 8c. One calculates the integral

68 $$B\left(x\right)=\int \frac{du}{I_{0}-x}=\int \frac{g\left(x\right)-g^{\prime }\left(x\right)x}{{g\left(x\right)}^{2}\left(I_{0}-x\right)}\;dx=-\frac{3}{2}ax\left(-4b+2I_{0}+x\right)+\left(c-3a{\left(b-I_{0}\right)}^{2}\right)\;\log\left[-I_{0}+x\right]$$

The result of eq 67 is

69 $$T_{R}=C_{0}\left[\left(B(x_{2}\right)-B\left(x_{1}\right)+B\left(x_{4}\right)-B\left(x_{3}\right)\right]$$

For the parameters of Figure 8 the period is *T*_*R*_ = 1.61*C*_0_ (in s), and the frequency of the relaxation oscillations in rad/s gives

70 $$\omega_{R}=\frac{2\pi }{T_{R}}=\frac{3.89}{C_{0}}$$

This is the blue dashed line in Figure 9b, that describes well the frequency of the oscillations at large *C*_0_.

### 4.3 Bifurcation and Oscillations at Constant Voltage

At fixed voltage *V*_*a*_, Figure 6d, the differential eq 45 becomes

71 $$\frac{du}{dt}=F=\frac{1}{C_{0}}\left[\frac{V_{a}}{R_{0}}-\left(\frac{1}{R_{0}}+g\left(x\right)\right)u\right]$$

The nullcline for *u̇* = *F* = 0 is

72 $$u=\frac{V_{a}}{1+g\left(x\right)R_{0}}$$

and for *ẋ* = *G* = 0 it is eq 48.

Again, the oscillation in Figure 10 occurs when the *u̇* = 0 nullcline (pink) intersects the intermediate branch of the S-shaped line *ẋ* = 0 (green) in the region where d*u*/d*x* < 0.

**Figure 10:**
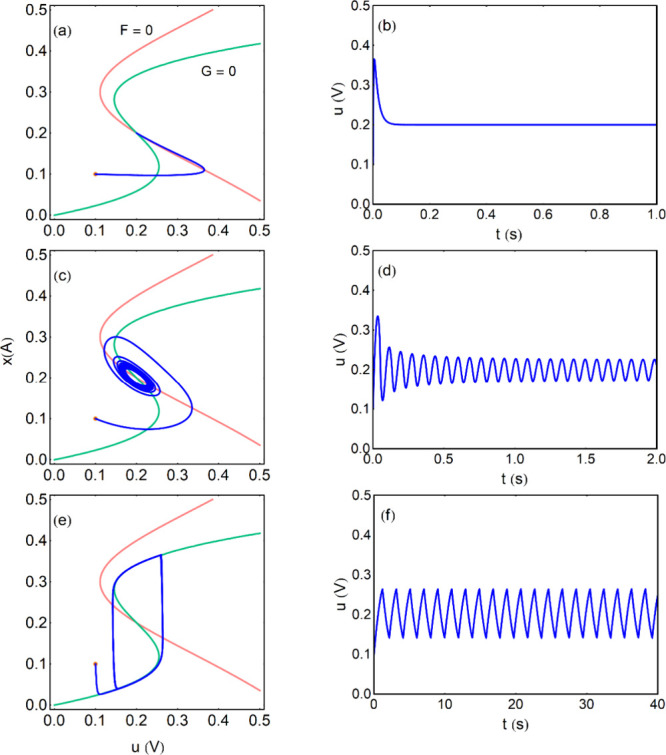
Oscillatory behavior at fixed voltage *V*_*a*_ = 1 *V*. Phase portrait plot, nullclines (a), (c), (e) and trajectory of *u* variable (b), (d), (f) of the dynamical evolution. The orange point is the initial condition. Parameters *a* = 50 *V/A* ^3^, *b* = 0.2 *A*,τ_k_ = 0.01 *s*, *R*_0_ = 4 Ω. (a, b) *C*_0_ = 0.001 *F*, ϵ = 2.5, where ϵ = τ_k_/τ_0_ and τ_0_ = *R*_0_ *C*_0_ (c, d) *C*_0_ = 0.0127 *F*, *T* = 0.0815 *s*, ϵ = 0.197, (e, f) *C*_0_ = 1 *F*, *T* = 1.95 *s*, ϵ = 2.5 × 10^–3^. The initial conditions are *u*(*t*_0_) = 0.1 V, *x*(*t*_0_) = 0.1 A.

The Jacobian matrix of the linearized system at steady-state is

73 $$J=\left[\begin{array}{ll} -\frac{1}{C_{0}}\left(\frac{1}{R_{0}}+g\left(x\right)\right) & -\frac{g^{\prime }\left(x\right)u}{C_{0}}\\ \frac{g\left(x\right)}{\tau_{k}} & \frac{1}{\tau_{k}}\left(g^{\prime }\left(x\right)u-1\right) \end{array}\right]$$

The trace is

74 $$T_{\lambda }=-\frac{1}{C_{0}}\left[\frac{1}{R_{0}}+g\left(x\right)\right]+\frac{1}{\tau_{k}}\left(g^{\prime }\left(x\right)u-1\right)$$

and the determinant

75 $$\Delta_{\lambda }=\frac{1}{{R_{0}C}_{0}\tau_{k}}\left[1-g^{\prime }\left(x\right)u+g\left(x\right)R_{0}\right]$$

The *T*_λ_ > 0 again requires that the conditions (*g*′(*x*)*u* – 1) > 0 and *g*(*x*) > 0 are satisfied. Thus, as it is well-known,^21–23^ the oscillations in S-shaped oscillators at fixed voltage *V*_*a*_ demand that the load orange line in Figure 6d intersects the unstable zone of the blue line. There are two parameters that can provide the match, *V*_*a*_ and *R*_0_. Therefore, the bifurcation parameter space is three-dimensional: (*V*_*a*_, *R*_0_, *C*_0_).

In Figure 11a the region of oscillation is obtained as a function of the bifurcation parameter *R*_0_, when *T*_λ_ > 0 since it is Δ_λ_ > 0 (continuous lines). The capacitor parameter at the bifurcation is

76 $$C_{0B}=\frac{1}{R_{0}}\frac{g\left(x\right)R_{0}+1}{g^{\prime }\left(x\right)u-1}\tau_{k}$$

It is shown in Figure 11b, with amplitude of the oscillations in Figure 11c.

**Figure 11:**
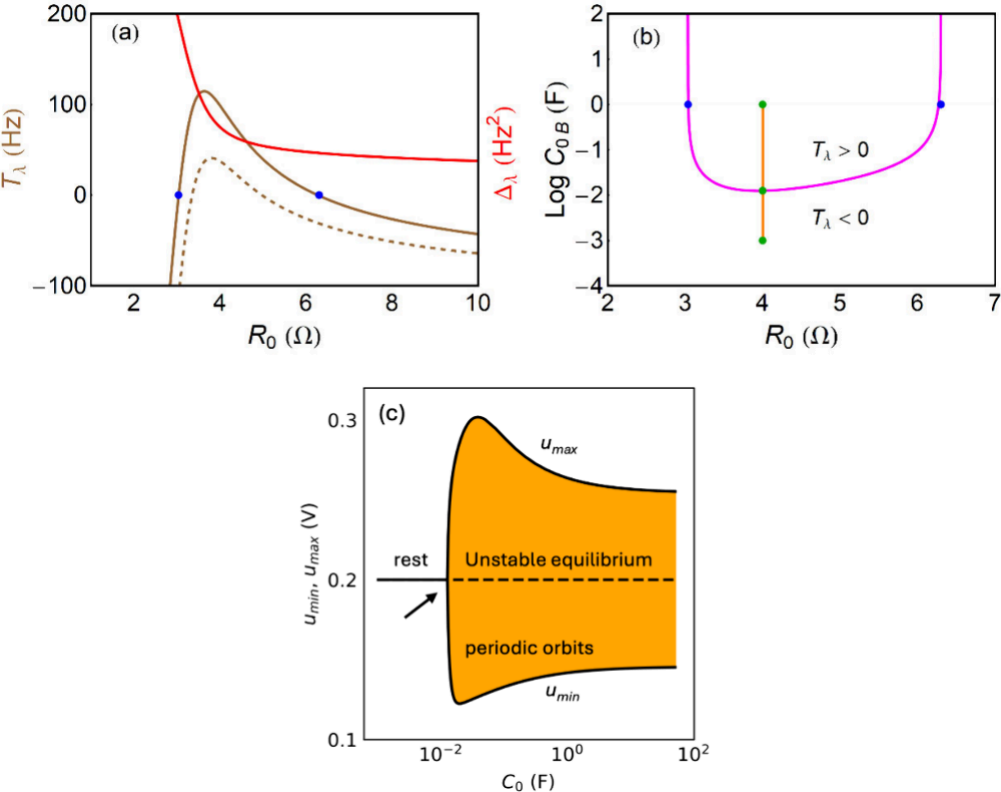
Bifurcation characteristics at constant voltage *V*_*a*_ = 1 *V*. (a) Trace and determinant of the Jacobian, as a function of bifurcation parameter *R*_0_. The blue points indicate the Hopf bifurcations at *R*_0*H*1_ = 3.03 Ω, *R*_0*H*2_ = 6.30 Ω and correspond to the folding points of the *u*(*I*_0_) curve. Line: *C*_0_ = 1 *F*, dashed line: *C*_0_ = 0.02 *F*. (b) Bifurcation diagram in the plane (*R*_0_, *C*_0_). The pink line is the value of capacitance that gives *T*_λ_ = 0. The orange line indicates the line of *C*_0_ through which calculation of the maximum and minimum *u* values has been performed in (c). The green points in the line indicate the *C*_0_ values in which the oscillations have been shown in Figure 10. (c). Calculated maximum and minimum *u* values. Parameters *a* = 50 *V/A* ^3^, *b* = 0.2 *A*, τ_k_ = 0.01 *s*.

The characteristic time of the external elements is τ_0_ = *R*_0_ *C*_0_. A parameter featuring the oscillatory properties is

77 $$\epsilon =\frac{\tau_{k}}{\tau_{0}}$$

Relaxation oscillations are obtained when ϵ → 0, as shown in Figure 10e. For pure relaxation oscillations (ϵ → 0) the bifurcation points correspond to the folding points, namely to the resistor values

78 $$R_{0H}=\frac{V_{a}-u\left(I_{F}\right)}{I_{F}}$$

Outside this region *T*_λ_ < 0 and the trajectory leads to a stable point.

However, the oscillation requires the slow/fast property in the two variables. In the dashed line of Figure 11a the *C*_0_ has been decreased, and the oscillatory region narrows, as this parameter approaches *C*_0B_. The orange line in Figure 11b establishes the ϵ_*B*_ where *T*_λ_(ϵ_*B*_) = 0. From eq 76 it follows that

79 $$\epsilon_{B}=\frac{g^{\prime }\left(x\right)u-1}{g\left(x\right)R_{0}+1}$$

Oscillations cannot happen when ϵ > ϵ_*B*_.

In Figure 10 the value of the capacitor is increased from top to bottom, following the orange line in Figure 11b. In Figure 10a, b the ϵ > ϵ_*B*_ and the system falls to the stable fixed point. In Figure 10b, c, the τ_0_ is small and ϵ = 0.125, close to the bifurcation limit ϵ_*B*_. These are the small amplitude, sinusoidal oscillations already described before. The frequency of oscillations at the Hopf bifurcation is

80 $$\omega_{0H}={\left[\frac{1}{{R_{0}C}_{0}\tau_{k}}\left(1-g^{\prime }\left(x\right)u+g\left(x\right)R_{0}\right)\right]}^{1/2}=\frac{1}{\tau_{k}}{\left[\left(g^{\prime }\left(x\right)u-1\right)\left(1-\frac{g^{\prime }\left(x\right)u}{1+g\left(x\right)R_{0}}\right)\right]}^{1/2}$$

In Figure 10e, f, relaxation oscillations are obtained. The period is given by the integral

81 $$T_{R}=\oint \frac{du}{F}=C_{0}R_{0}\oint \frac{du}{V_{a}-\left(1+{g\left(x\right)R}_{0}\right)u}$$

Using the same method as above, for the parameters of Figure 10 the calculation of the period gives *T*_*R*_ = 0.41τ_0_. Thus, τ_0_ sets the time scale of the relaxation oscillations at ϵ ≪ 1. The frequency of the relaxation oscillations is

82 $$\omega_{R}=\frac{2\pi }{T_{R}}=\frac{15.2}{\tau_{0}}$$

Methods to obtain the oscillation frequency outside of the relaxation limit by asymptotic expansion have been amply explored.^92,105,106^

## 5 Applications

### 5.1 Binary Oxide Oscillators

The archetypal S-type oscillator is formed by substoichiometric transition metal oxides sandwiched between two metal electrodes, as VO_2_,^7,9,107^ NbO_2_ ^108^ and TaO_*x*_.^6^ The negative S-type resistance in binary oxides memristors is associated with the insulator–metal transition (IMT) caused by temperature-dependent transport effects.^94–99^ These physical mechanisms leading to oscillatory behaviors have been reviewed^49–51^ and many studies of binary oxide oscillators have been developed.^95,98,109–111^

Figure 12 shows the characteristic features of binary oxide oscillators.^109^ In the measurement at constant current the negative resistance feature is well obtained in Figure 12b. On the other hand, by cycling the voltage the system jumps at the folding points of the *u*(*I*) (voltage–current) curve. This cycling behavior is the essence of the relaxation oscillations. The feature is also known as “threshold switching” ^98,99,109^ and it is widely used to make artificial neurons.^27–29^

**Figure 12:**
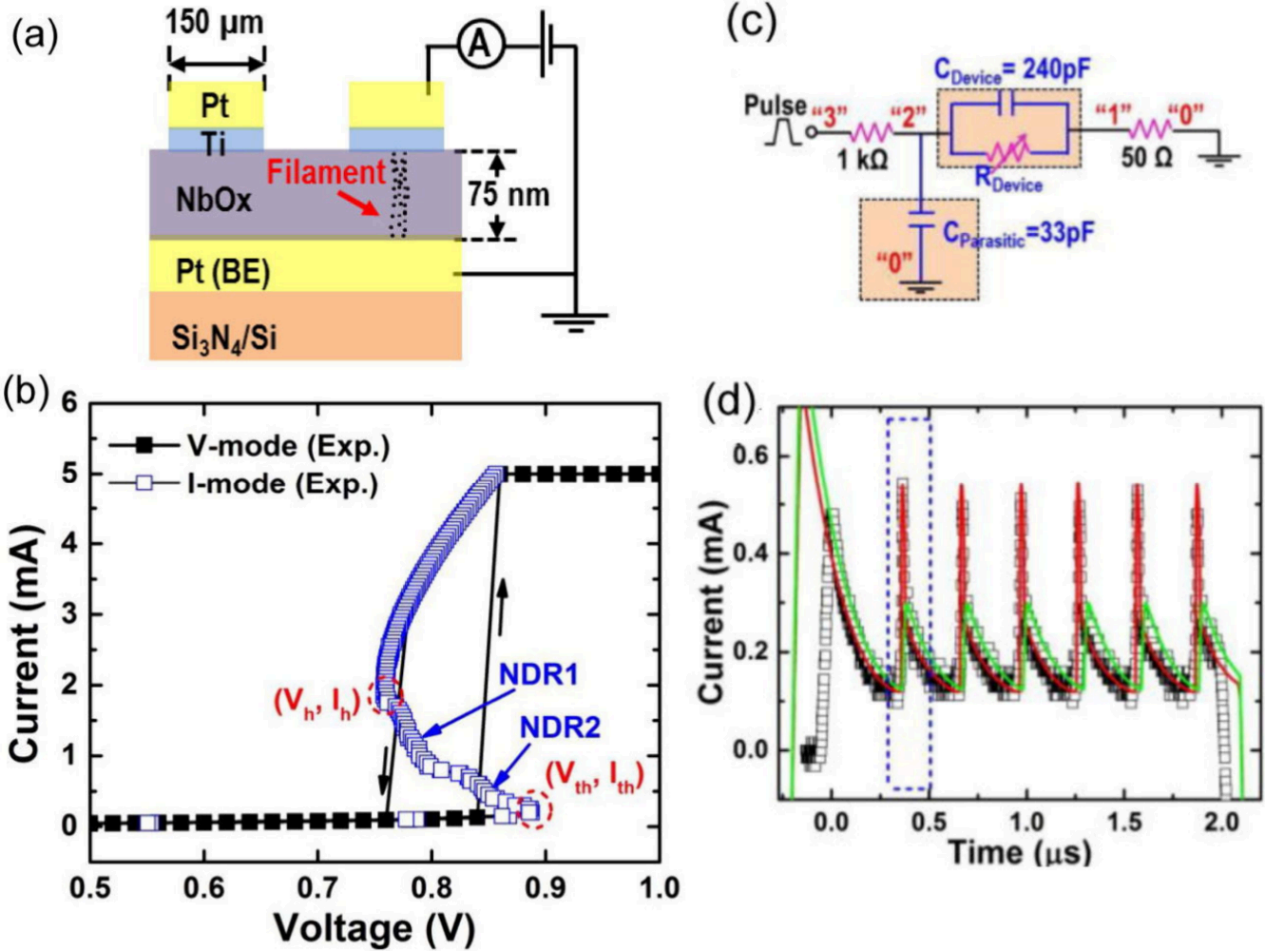
(a) Schematic of Pt/Ti/NbO_*x*_/Pt test devices and the measuring conditions. (b) Measured curves for both voltage- and current-sweeping modes, showing a clear CC-NDR characteristic with multi-NDR properties. (c) Schematic of the electrical circuit used in simulation to study the dynamics of self-oscillations. (d) Measured (black squares) and simulated (red curves) oscillation waveforms of the current through the 50 Ω resistor for a source voltage of *V*_*s*_ = 1.2 V (2 μs) and the series resistance of *R*_*L*_ = 1 kΩ. Reprinted from ref 109 with the permission of AIP Publishing.^109^

Recently, control of spiking frequencies has been discussed.^112^ Figure 13b and c show the modification of oscillations of a VO_2_ oscillator circuit by the change of the external capacitor. Figure 13d shows the evolution of the frequency of oscillations as a function of capacitance.

**Figure 13:**
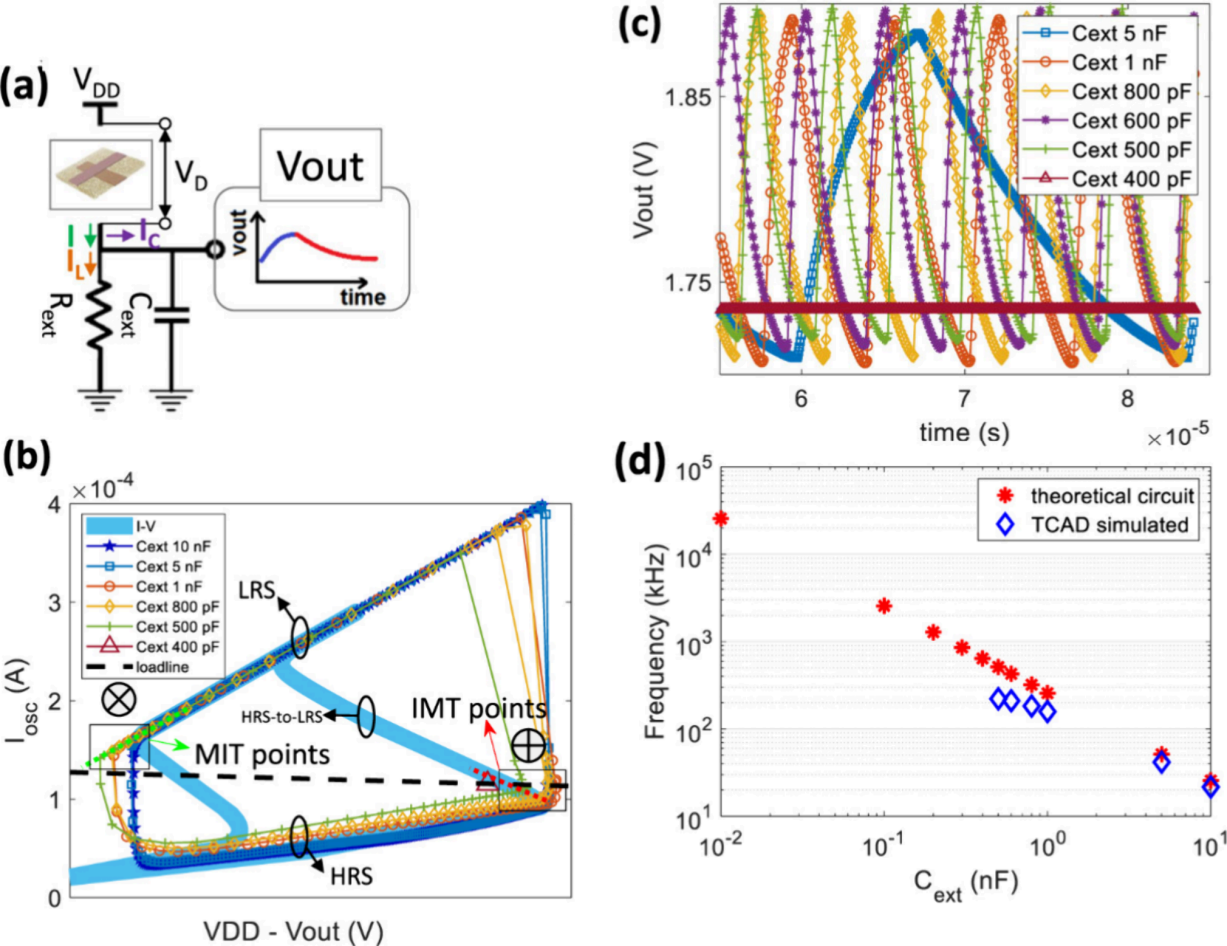
(a) Schematics of a VO_2_ oscillator circuit. I, I_L_ and I_C_ are the currents across the VO_2_ device, *R*_*ext*_ and *C*_*ext*_, respectively. Representative, nonsinusoidal waveform of voltage at the output node over one period is also shown. (b) Curves of I versus *V*_*D*_ = *V*_*DD*_ – *V*_*out*_ (symbols) as extracted from simulated oscillations (10 nF–400 pF). For comparison, quasi-static I versus *V*_*D*_ (azure thick line) is also shown. (c) Electrothermal 3D TCAD mixed-mode simulations of voltages at the output node *V*_*out*_ for *C*_*ext*_ in the interval 5 nF–400 pF. (d) Frequency vs *C*_*ext*_. Blue-diamond curve is from electrothermal 3D TCAD mixed-mode simulated oscillations. In this case, the frequency of oscillations ranges from 41.7 kHz (5 nF) up to 221.4 kHz (500 pF). At 400 pF, the oscillations are inhibited. Red-asterisk curve is obtained by theoretical calculation through a purely circuital model of the VO_2_ oscillator where the VO_2_ device is simplified as a two-state VO_2_ variable resistor whose RS is activated by threshold voltages. Reprinted with permission from ref 8 licensed under a Creative Commons Attribution (CC BY 4.0) license.

### 5.2 Electrochemical Oscillators

The appearance of electrochemical oscillations,^21,37,38,42^ requires a fast positive feedback mechanism and a slow inhibitor one. The positive feedback variable is typically the electrode potential (*E*) or the surface coverage of adsorbed species (θ), depending on the system. In many cases, an increase in electrode potential accelerates a reaction, which in turn further increases the potential, creating a self-reinforcing loop. This is commonly observed in passivation and electrodissolution reactions, where the formation of an oxide layer or intermediate species alters the charge transfer rate. Similarly, in electrocatalytic reactions such as CO oxidation on platinum, the surface coverage of reactive intermediates can exhibit positive feedback. A small increase in the coverage of adsorbed species can enhance their production rate, leading to further accumulation and self-reinforcement. This type of feedback is evident in systems like metal electrodissolution, where chloride adsorption and oxide formation play a role, or in electrocatalytic oxidation, where intermediates influence reaction kinetics.

Most electrochemical systems yield differential negative resistances of type N (N-NDR) in the *IV* characteristics,^89^ being their complementary counterparts, the S-type systems (S-NDR), the exception. Because of the intrinsic *IV* shape, such a fast positive mechanism is driven by the working electrode potential in N-NDR systems while in S-type systems the electrode potential plays the role of the slow inhibitor. Therefore, an extremely fast autocatalytic chemical reaction is needed to provide the necessary positive feedback, although similar effects could be achieved by large values of the double electrode layer capacitance. This is the reason why it is difficult to find S-type systems in this field and few examples have been reported in the literature. Perhaps, the most representative one corresponds to the electro-crystallization of Zn,^42,84,104,113^ see Figure 7b and Figure 14. Other cases involve surface phase transitions of organic adsorbates, for instance in the periodate reduction on Au(111) single crystal electrodes in the presence of camphor^114^ and CO bulk oxidation.^115^

**Figure 14:**
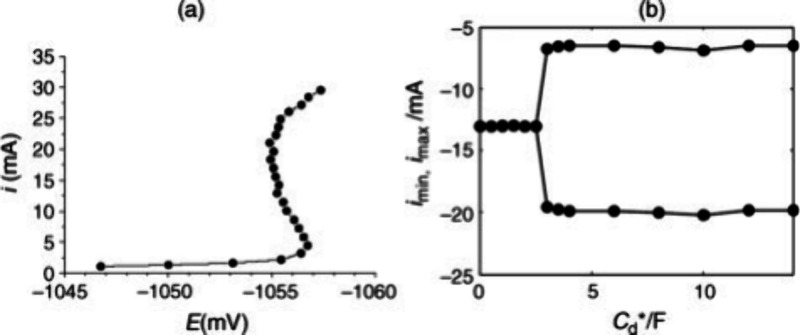
Bistability and oscillations in S-NDR Zn electrodeposition system. (a) IR-compensated (*R*_*s*_ = 3.0 Ω) S-shaped polarization curve of Zn electrodeposition. Experimental conditions include 7 mm diameter Zn disk electrode rotated at 1000 rpm in 0.72 mol dm^–3^ ZnCl_2_ + 2.67 mol dm^–3^ NH_4_Cl buffer (pH = 5.2) at 26 °C, scan rate of −4.0 mVs^–1^. (b) One-parameter bifurcation diagram of Zn electrodeposition at V_0_ = −1100 mV and R = 9.7 Ω, showing the minima and maxima of current oscillations as a function of the pseudocapacitance C_d_*. Reproduced from ref 42 with permission from John Wiley and Sons. Copyright 2011.^42^

## 6 Discussion and Conclusion

The oscillation of the S-type oscillator is controlled by several features. The stationary current must be in a domain of negative resistance, which is obtained at constant current, or with a matched resistance at constant voltage. This requirement introduces the constraint that the current must occur between the two folding points of the *u*(*I*) curve.

In addition, for a given current (or voltage) there is a minimum value of the capacitor, *C*_0B_, at which the Hopf bifurcation occurs. Close to this value, the Hopf theorem ensures the conditions for existence of a limit cycle^116,117^ where the oscillations have small amplitude and nearly sinusoidal form. In the other extreme, the capacitor becomes large, and the oscillations slow down. Relaxation oscillations are obtained, where the frequency is independent of the relaxation time of the internal variable *x*, since this variable becomes very fast, and the oscillations are controlled by the charge and discharge of the external capacitor between the folding points of the current–voltage. For the fixed voltage oscillator, the period of oscillations is of the order τ_0_ = *R*_0_ *C*_0_.

These conclusions are fairly general. For any two-contact system controlled by a single internal variable and showing the S-shape of the current–voltage, the circuit of Figure 6a produces oscillations that change from sinusoidal to relaxation, according to the ratio of the external *R*_0_ *C*_0_ elements to the internal relaxation time of the nonlinear element, τ_*k*_. Here the simplest oscillator configuration of Figure 6a, with two differential equations, was explored. Including more circuit elements, or combining several nonlinear oscillators, the oscillations can be further modulated for biologically plausible spiking patterns.^107^

In conclusion, a general model was introduced for an oscillator with a single degree of freedom and its oscillatory properties were analyzed, ranging from harmonic oscillations near the Hopf bifurcation point to slower relaxation oscillations. The dependence of frequency and period on circuit parameters has been explicitly derived. This classification of behaviors in single-variable S-type oscillators provides a foundation for practical investigation and application of these systems in neuromorphic circuits.

## Data Availability

The data presented here can be accessed at 10.5281/zenodo.14711801 (Zenodo) under the license CC-BY-4.0 (Creative Commons Attribution-ShareAlike 4.0 International).
